# Extremophiles and Extreme Environments

**DOI:** 10.3390/life3030482

**Published:** 2013-08-07

**Authors:** Pabulo Henrique Rampelotto

**Affiliations:** Interdisciplinary Center for Biotechnology Research, Federal University of Pampa, Antônio Trilha Avenue, P.O.Box 1847, 97300-000, São Gabriel–RS, Brazil; E-Mail: pabulo@lacesm.ufsm.br

Over the last decades, scientists have been intrigued by the fascinating organisms that inhabit extreme environments. Such organisms, known as extremophiles, thrive in habitats which for other terrestrial life-forms are intolerably hostile or even lethal. They thrive in extreme hot niches, ice, and salt solutions, as well as acid and alkaline conditions; some may grow in toxic waste, organic solvents, heavy metals, or in several other habitats that were previously considered inhospitable for life. Extremophiles have been found depths of 6.7 km inside the Earth’s crust, more than 10 km deep inside the ocean—at pressures of up to 110 MPa; from extreme acid (pH 0) to extreme basic conditions (pH 12.8); and from hydrothermal vents at 122 °C to frozen sea water, at −20 °C. For every extreme environmental condition investigated, a variety of organisms have shown that they not only can tolerate these conditions, but that they also often require those conditions for survival. 

They are classified according to the conditions in which they grow: As thermophiles and hyperthermophiles (organisms growing at high or very high temperatures, respectively), psychrophiles (organisms that grow best at low temperatures), acidophiles and alkaliphiles (organisms optimally adapted to acidic or basic pH values, respectively), barophiles (organisms that grow best under pressure), and halophiles (organisms that require NaCl for growth). In addition, these organisms are normally polyextremophiles, being adapted to live in habitats where various physicochemical parameters reach extreme values. For example, many hot springs are acid or alkaline at the same time, and usually rich in metal content; the deep ocean is generally cold, oligotrophic (very low nutrient content), and exposed to high pressure; and several hypersaline lakes are very alkaline.

Extremophiles may be divided into two broad categories: extremophilic organisms which require one or more extreme conditions in order to grow, and extremotolerant organisms which can tolerate extreme values of one or more physicochemical parameters though growing optimally at “normal” conditions. 

Extremophiles include members of all three domains of life, *i.e.*, bacteria, archaea, and eukarya. Most extremophiles are microorganisms (and a high proportion of these are archaea), but this group also includes eukaryotes such as protists (e.g., algae, fungi and protozoa) and multicellular organisms.

Archaea is the main group to thrive in extreme environments. Although members of this group are generally less versatile than bacteria and eukaryotes, they are generally quite skilled in adapting to different extreme conditions, holding frequently extremophily records. Some archaea are among the most hyperthermophilic, acidophilic, alkaliphilic, and halophilic microorganisms known. For example, the archaeal *Methanopyrus kandleri* strain 116 grows at 122 °C (252 °F, the highest recorded temperature), while the genus *Picrophilus* (e.g., *Picrophilus torridus*) include the most acidophilic organisms currently known, with the ability to grow at a pH of 0.06.

Among bacteria, the best adapted group to various extreme conditions is the cyanobacteria. They often form microbial mats with other bacteria, from Antarctic ice to continental hot springs. Cyanobacteria can also develop in hypersaline and alkaline lakes, support high metal concentrations and tolerate xerophilic conditions (*i.e.*, low availability of water), forming endolithic communities in desertic regions. However, cyanobacteria are rarely found in acidic environments at pH values lower than 5–6. 

Among eukaryotes, fungi (alone or in symbiosis with cyanobacteria or algae forming lichens) are the most versatile and ecologically successful phylogenetic lineage. With the exception of hyperthermophily, they adapt well to extreme environments. Fungi live in acidic and metal-enriched waters from mining regions, alkaline conditions, hot and cold deserts, the deep ocean and in hypersaline regions such as the Dead Sea. Nevertheless, in terms of high resistance to extreme conditions, one of the most impressive eukaryotic polyextremophiles is the tardigrade, a microscopic invertebrate. Tardigrades can go into a hibernation mode, called the tun state, whereby it can survive temperatures from −272 °C (1 °C above absolute zero!) to 151 °C, vacuum conditions (imposing extreme dehydration), pressure of 6,000 atm as well as exposure to X-rays and gamma-rays. Furthermore, even active tardigrades show tolerance to some extreme environments such as extreme low temperature and high doses of radiation.

In general, the phylogenetic diversity of extremophiles is high and very complex to study. Some orders or genera contain only extremophiles, whereas other orders or genera contain both extremophiles and non-extremophiles. Interestingly, extremophiles adapted to the same extreme condition may be broadly dispersed in the phylogenetic tree of life. This is the case for different psychrophiles or barophiles, for which members may be found dispersed in the three domains of life. There are also groups of organisms belonging to the same phylogenetic family that have adapted to very diverse extreme or moderately extreme conditions.

Over the last few decades, the fast development of molecular biology techniques has led to significant advances in the field, allowing us to investigate intriguing questions on the nature of extremophiles with unprecedented precision. In particular, new high-throughput DNA sequencing technologies have revolutionized how we explore extreme microbiology, revealing microbial ecosystems with unexpectedly high levels of diversity and complexity. Nevertheless, a thorough knowledge of the physiology of organisms in culture is essential to complement genomic or transcriptomic studies and cannot be replaced by any other approach. Consequently, the combination of improved traditional methods of isolation/cultivation and modern culture-independent techniques may be considered the best approach towards a better understanding of how microorganisms survive and function in such extreme environments. 

Based on such technological advances, the study of extremophiles has provided, over the last few years, ground-breaking discoveries that challenge the paradigms of modern biology and make us rethink intriguing questions such as “what is life?”, “what are the limits of life?”, and “what are the fundamental features of life?”. These findings have made the study of life in extreme environments one of the most exciting areas of research, and can tell us much about the fundaments of life.

The mechanisms by which different organisms adapt to extreme environments provide a unique perspective on the fundamental characteristics of biological processes, such as the biochemical limits to macromolecular stability and the genetic instructions for constructing macromolecules that stabilize in one or more extreme conditions. These organisms present a wide and versatile metabolic diversity coupled with extraordinary physiological capacities to colonize extreme environments. In addition to the familiar metabolic pathway of photosynthesis, extremophiles possess metabolisms based upon methane, sulfur, and even iron. 

Although the molecular strategies employed for survival in such environments are still not fully clarified, it is known that these organisms have adapted biomolecules and peculiar biochemical pathways which are of great interest for biotechnological purposes. Their stability and activity at extreme conditions make them useful alternatives to labile mesophilic molecules. This is particularly true for their enzymes, which remain catalytically active under extremes of temperature, salinity, pH, and solvent conditions. Interestingly, some of these enzymes display polyextremophilicity (*i.e.,* stability and activity in more than one extreme condition) that make their wide use in industrial biotechnology possible.

From an evolutionary and phylogenetic perspective, an important achievement that has emerged from studies involving extremophiles is that some of these organisms form a cluster on the base of the tree of life. Many extremophiles, in particular the hyperthermophiles, lie close to the “universal ancestor” of all organisms on Earth. For this reason, extremophiles are critical for evolutionary studies related to the origins of life. It is also important to point out that the third domain of life, the archaea, was discovered partly due to the first studies on extremophiles, with profound consequences for evolutionary biology.

Furthermore, the study of extreme environments has become a key area of research for astrobiology. Understanding the biology of extremophiles and their ecosystems permits developing hypotheses regarding the conditions required for the origin and evolution of life elsewhere in the universe. Consequently, extremophiles may be considered as model organisms when exploring the existence of extraterrestrial life in planets and moons of the Solar System and beyond. For example, the microorganisms discovered in ice cores recovered from the depth of the Lake Vostok and other perennially subglacial lakes from Antarctica may serve as models for the search of life in the Jupiter’s moon Europa. Microbial ecosystems found in extreme environments like the Atacama Desert, the Antarctic Dry Valleys and the Rio Tinto may be analogous to potential life forms adapted to Martian conditions. Likewise, hyperthermophilic microorganisms present in hot springs, hydrothermal vents and other sites heated by volcanic activity in terrestrial or marine areas may resemble potential life forms existing in other extraterrestrial environments. Recently, the introduction of novel techniques such as Raman spectroscopy into the search of life signs using extremophilic organisms as models has open further perspectives that might be very useful in astrobiology.

With these groundbreaking discoveries and recent advances in the world of exthemophiles, which have profound implications for different branches of life sciences, our knowledge about the biosphere has grown and the putative boundaries of life have expanded. However, despite the latest advances we are just at the beginning of exploring and characterizing the world of extremophiles. This special issue discusses several aspects of these fascinating organisms, exploring their habitats, biodiversity, ecology, evolution, genetics, biochemistry, and biotechnological applications in a collection of exciting reviews and original articles written by leading experts and research groups in the field. I would like to thank the authors and co-authors for submitting such interesting contributions. I also thank the Editorial Office and numerous reviewers for their valuable assistance in reviewing the manuscripts.

